# A Naturally Derived Carrier for Photodynamic Treatment of Squamous Cell Carcinoma: In Vitro and In Vivo Models

**DOI:** 10.3390/pharmaceutics12060494

**Published:** 2020-05-29

**Authors:** Soad Nasr, Mai Rady, Aya Sebak, Iman Gomaa, Walid Fayad, Menna El Gaafary, Mahmoud Abdel-Kader, Tatiana Syrovets, Thomas Simmet

**Affiliations:** 1 Institute of Pharmacology of Natural Products & Clinical Pharmacology, Ulm University, D-89081 Ulm, Germany; soad.nasr@aucegypt.edu (S.N.); mennat_elgaafary@yahoo.com (M.E.G.); 2 Department of Chemistry, School of Sciences and Engineering, The American University in Cairo (AUC), Cairo 11835, Egypt; 3 Pharmaceutical Technology Department, Faculty of Pharmacy and Biotechnology, German University in Cairo (GUC), New Cairo City 11865, Egypt; mai.rady@guc.edu.eg (M.R.); aya.sebak-mohamed@guc.edu.eg (A.S.); 4 Biochemistry Department, Faculty of Pharmacy, October University for Modern Sciences and Arts (MSA), 6th of October City 12573, Egypt; igomaa@msa.eun.eg; 5 Drug Bioassay-Cell Culture Laboratory, Pharmacognosy Department, National Research Centre, Dokki, Giza 12622, Egypt; walidfayyad@gmail.com; 6 Department of Pharmacognosy, College of Pharmacy, Cairo University, Cairo 11562, Egypt; 7 National Institute of Laser Enhanced Sciences (NILES), Cairo University (CU), Giza 12511, Egypt

**Keywords:** drug carrier, chlorophyll derivatives, photodynamic therapy, squamous cell carcinoma, singlet oxygen, reactive oxygen species, apoptosis, 3D spheroid cell culture, tumor xenograft, chick chorioallantoic membrane assay

## Abstract

Photodynamic therapy (PDT) is a non-invasive treatment strategy that includes the combination of three components—a photosensitizer, a light source, and tissue oxygen. PDT can be used for the treatment of skin diseases such as squamous cell carcinoma. The photosensitizer used in this study is the naturally derived chlorophyll derivative chlorin e6 (Ce6), which was encapsulated in ultradeformable ethosomes. Singlet oxygen production by Ce6 upon laser light irradiation was not significantly affected by encapsulation into ethosomes. PDT of squamous cell carcinoma cells treated with Ce6 ethosomes triggered increased mitochondrial superoxide levels and increased caspase 3/7 activity, resulting in concentration- and light-dose-dependent cytotoxicity. Ce6 ethosomes showed good penetration into 3D squamous cell carcinoma spheroids, which upon laser light irradiation exhibited reduced size, proliferation, and viability. The PDT effect of Ce6 ethosomes was specific and showed higher cytotoxicity against squamous cell carcinoma spheroids compared to normal skin fibroblast spheroids. In addition, PDT treatment of squamous cell carcinoma xenografts grown on chorioallantoic membranes of chick eggs (CAM) exhibited reduced expression of Ki-67 proliferation marker and increased terminal deoxynucleotidyl transferase dUTP nick end labelling (TUNEL) staining, indicating reduced proliferation and activation of apoptosis, respectively. The results demonstrate that Ce6-loaded ethosomes represent a convenient formulation for photodynamic treatment of squamous cell carcinoma.

## 1. Introduction

Non-melanoma skin cancers are the most common types of skin malignancies in Caucasians. They include squamous cell carcinoma, the second most common type of skin cancer after basal cell carcinoma [1,2]. Most squamous cell carcinoma occur in sun-exposed areas of the skin, characterized by an uncontrolled growth of squamous cells that lie mostly in the epidermal cell layer of the skin, but which can metastasize deeper in advanced cancer stages [3].

Photodynamic therapy (PDT) is a non-invasive therapy based on the activation of a light-sensitive drug—a photosensitizer—by light of a specific wavelength. In the presence of tissue oxygen, reactive oxygen species such as singlet oxygen (^1^O_2_) are generated and interact with cellular components, such as proteins, lipids, and DNA, causing cell death [4]. Since PDT is a light-triggered process, the photodynamic reaction can be restricted to the light-exposed area; thus, it is safer than chemotherapy [4]. PDT is used in a wide range of therapeutic fields, especially in cancer, where selective production of ^1^O_2_ in target tissue and preferential accumulation of photosensitizer (particularly when incorporated into larger carrier particles) in tumors through enhanced permeation and retention effect (EPR) add to its specificity [4,5,6,7].

Chlorins are a class of porphyrin-based photosensitizers used in PDT [8]. Some chlorin family photosensitizers have already been approved by the FDA, such as Foscan^®^, Photofrin^®^, and Visudyne^®^ [9]. Chlorin e6 (Ce6) is a second-generation, FDA-approved photosensitizer (FDA UNII: 5S2CCF3T1Z) with improved pharmacokinetic properties. Ce6 has shown promising therapeutic properties and has been approved as N-aspartyl chlorin e6 in Japan to treat lung cancer [9]. Ce6 is made of a tetrapyrrole ring that is structurally related to porphyrins but has a higher degree of saturation of the ring system. Ce6 can be excited at a wavelength range of 620–660 nm, depending on the medium pH, thus significantly improving the penetration depth of PDT in vivo [10]. In addition, due to the strong fluorescence emission of Ce6 in the red region, it can serve as an imaging agent for theranostic approaches [6]. However, the hydrophobicity of Ce6 might restrict its clinical use [8,10]. Therefore, encapsulation of the drug into biocompatible carriers such as polymers, liposomes, and inorganic nanoparticles has been investigated to enhance its therapeutic efficacy [6,11,12,13].

Topical drug delivery in PDT treatment of skin cancer constitutes a comfortable and safe treatment option in comparison to systemically active drugs. However, the stratum corneum, the outermost layer of the skin, serves as a barrier for limiting drug absorption via topical delivery. Nano- and microvesicles are currently being used for the effective delivery of drugs through the skin due to the increased surface area of the particles, which enhances skin penetration [14,15,16]. Ethosomes are carrier vesicle systems consisting of phospholipids, alcohol, surfactant, and water. The combination of alcohol and soft phospholipid vesicles in ethosomes yields ultradeformable properties and allows for greater penetration into deeper skin layers [15,16]. In our previous study, the photosensitizer ferrous chlorophyllin loaded into ethosomes exhibited higher skin penetration compared to ferrous chlorophyllin loaded into lipid-coated chitosan particles, despite the fact that the latter particles were half the size of ethosomes [17].

Although cell monolayers are frequently used for in vitro studies, such models have various limitations. The 3D culture models provide a microenvironment that more closely mimics the environment found in tumor tissue [18]. The existing in vivo models for squamous cell carcinoma have restrictions in mimicking some crucial tumorigenic phenotypes, such as invasion, because the basement membrane of the surface epithelium is bypassed in mouse models [19]. In this study, the in ovo chick chorioallantoic membrane assay model (CAM) was used as an in vivo xenograft model for human squamous cell carcinoma, where cells were seeded onto the chorioallantoic membrane to allow formation of tumor xenografts and their invasion into a highly vascularized membrane. The chorioallantoic membrane is made mainly of type IV collagen, which mimics the basement membrane of the human epithelium. CAM models have been studied to evaluate anticancer drug effects on tumor growth and invasion, including effects on melanoma and head and neck squamous cell carcinoma [19,20,21,22,23,24].

So far, surgical treatment still remains the most effective technique for squamous cell carcinoma. Other treatment approaches such as radiation therapy, cryosurgery, and topical ointments are also occasionally used [3]. In the current study, the application of naturally derived lipid for a facile fabrication of the highly hydrophobic photosensitizer Ce6 is investigated. This is the first study to employ Ce6 ethosomes to evaluate their ^1^O_2_ production, apoptosis, and PDT effect on the skin squamous cell carcinoma monolayer, 3D spheroids, and in vivo on squamous cell carcinoma xenografts in the CAM model.

## 2. Materials and Methods

### 2.1. Materials

Human squamous cell carcinoma, A431 (ACC 91, DSMZ German collection of microorganisms and cell cultures, Braunschweig, Germany), normal human skin fibroblasts, BJ (American Type Culture Collection, ATCC^®^ CRL-2522™, Manassas, VI, USA), lecithin from soybean 90% (PanReac AppliChem, Darmstadt, Germany), chlorin e6 (Cayman Chemical, Ann Arbor, MI, USA), cremophor A25 (Sigma-Aldrich, Taufkirchen, Germany), anthracene-9,10-dipropionic acid disodium salt (ADPA) (Santa Cruz Biotechnology, Santa Cruz, CA, USA), sodium azide (99%) (Loba Chemie, Mumbai, India) were obtained as indicated. In addition, Dulbecco’s modified Eagle’s medium/nutrient Mixture F-12 (DMEM/F12), fetal bovine serum (FBS), trypsin–EDTA, phosphate-buffered saline (PBS), penicillin-streptomycin mixture (10,000 U/mL penicillin, 10,000 µg/mL streptomycin), and trypan blue 0.4% in 0.85% NaCl (Lonza, Basel, Switzerland) were obtained as indicated. Antihuman proliferation antigen Ki-67 (DakoCytomation, Glostrup, Denmark), TUNEL assay (terminal deoxynucleotidyl transferase-mediated dUTP nick-end labelling) (Roche, Basel, Switzerland), and hematoxylin eosin stain (HE, Sigma-Aldrich) were purchased as designated. MTT [3-(4,5-dimethylthiazol-2-yl)-2,5-diphenyltetrazolium bromide] (Serva, Heidelberg, Germany), poly-2-hydroxyethyl methacrylate (poly-HEMA, Sigma-Aldrich), p-nitrophenylphosphate (Pierce Biotechnology Inc., Rockford, IL, USA), non-essential amino acids (NEAA), matrigel (BD Biosciences, San Jose, CA, USA), Z-DEVD-R110 caspase 3/7 substrate (Bachem, Bubendorf, Switzerland), and MitoSOX Red (Molecular Probes, Eugene, OR, USA) were purchased from the respective suppliers.

### 2.2. Formulation of Chlorin-E6-Loaded Ethosomes (Ce6 Ethosomes)

Ce6 ethosomes were prepared using the cold method according to Touitou et al. [16] with some modification. Ce6 powder (5 mg), 250 mg lecithin from soybean, and 10 mg cremophor-A25 were added to ethanol (1.5 mL, 20% *v*/*v*) and mixed in a closed vessel at 30 °C. Ultrapure water (3.5 mL) was slowly added dropwise to the above mixture with continuous stirring at 700 rpm for 30 min. The mixture was then sonicated in a water bath (UC-20500B Chrom Tech, Taipei, Taiwan) at maximum power for 30 min. The resulting vesicular suspension was cooled down to room temperature (25 ± 1 °C) for 10 min then stored at 4 °C.

### 2.3. Physicochemical Characterization of Ce6 Ethosomes

Dynamic light scattering and electrophoretic mobility analysis were used to determine the mean size and surface charge of Ce6 ethosomes, respectively, by using a Zetasizer Nano ZS (Malvern Instruments ZS, Malvern, UK). A 1:10 dilution yielding an end concentration of 0.16 mM Ce6 ethosomes was prepared in ultrapure water and measured to assess the size and surface charge of the ethosomes. Ce6 ethosome molarity specifications refer to the amount of incorporated Ce6. For transmission electron microscopy (TEM), Ce6 ethosomes were stained with 1% (*w*/*w*) phosphotungstic acid and dried on a copper grid before TEM imaging (HR Jeol 2100, JEOL Ltd., Tokyo, Japan).

To determine the amount of photosensitizer encapsulated in the carriers, Ce6 ethosomes were 22,000× *g* at 4 °C for 90 min. The supernatant was separated and its absorbance was measured spectrophotometrically at Ce6 λ_max_ = 405 nm. A calibration curve of Ce6 was plotted by dissolving 1 mg of Ce6 in 1 mL dimethyl sulfoxide (DMSO), then diluted with ultrapure water to prepare a stock solution at a concentration of 15 µg/mL. Using serial dilutions, concentrations of 0.01, the 0.05, 0.1, 0.2, and 0.3 µg/mL solutions were obtained and their absorbance was measured by a UV-Vis spectrophotometer (Jasco Corporation, Tokyo, Japan) to determine absorption at λ_max_. The following equations were used to calculate the entrapment efficiency (EE) and the drug loading (DL) of the photosensitizer [25].

$$\mathrm{EE}\;\left(\%\right)=\frac{\mathrm{Total}\;\mathrm{amount}\;\mathrm{of}\;\mathrm{drug}\;-\mathrm{Amount}\;\mathrm{of}\;\mathrm{free}\;\mathrm{drug}}{\mathrm{Total}\;\mathrm{amount}\;\mathrm{of}\;\mathrm{drug}}\times 100$$

$$\mathrm{DL}\;\left(\%\right)=\frac{\mathrm{Total}\;\mathrm{amount}\;\mathrm{of}\;\mathrm{drug}\;-\mathrm{Amount}\;\mathrm{of}\;\mathrm{free}\;\mathrm{drug}}{\mathrm{Amount}\;\mathrm{of}\;\mathrm{carrier}\;}\times 100$$

### 2.4. Evaluation of Singlet Oxygen (^1^O_2_) Production

Anthracene-9,10-dipropionic acid disodium salt (ADPA) was used as a singlet oxygen (^1^O_2_) sensor. At the excitation wavelength of 378 nm, ADPA displays two strong fluorescence emission bands between 400 and 450 nm. However, in the presence of ^1^O_2_, ADPA is bleached into an endoperoxide, a non-fluorescent product. As a result, the decrease of ADPA fluorescence could be monitored as an indicator of ^1^O_2_ generation [26]. Fluorescence measurements for the detection of ^1^O_2_ generation for Ce6 and Ce6 ethosomes was carried out using a multiwell plate reader (Victor 3V 1420, Perkin Elmer, Waltham, MA, USA) at 450 nm. Solutions of Ce6 and Ce6 ethosomes (0.3 µM, each) were prepared in ultrapure water and were mixed at a ratio of 1:1 with 12.5 mM of ADPA dissolved in 20% methanol. The samples were irradiated with a laser source (652 ± 5 nm) for 0, 1, 2, 3, 4, and 5 min at a power density of 200 mW/cm^2^ (1091-G diode laser, Biolitec Biomedical Technology, Jena, Germany); the above values are equivalent to 12, 24, 36, 48, and 60 J/cm^2^, respectively. Negative control samples that ensured that the decrease in fluorescence is due to ^1^O_2_ production additionally contained 5 × 10^−2^ M sodium azide (NaN_3_) as an ^1^O_2_ quencher. Light controls contained ADPA and solvent and were irradiated with the same light exposure as samples. Dark controls were identical to samples and were analyzed at the same time points but in the absence of laser irradiation.

### 2.5. Analysis of Cell Viability

The light doses used in this study were 12 J/cm^2^ and 60 J/cm^2^. Higher doses have previously been shown to be cytotoxic [17]. Squamous cell carcinoma cells (1 × 10^3^ cells/well) were seeded in a 96-well plate and incubated overnight under standard culture conditions (37 °C, 5% CO_2_). Ce6 ethosomes were diluted to the desired concentration in DMEM complete medium and incubated with the cells for additional 24 h. Thereafter, cells were irradiated with a monochromatic red laser (652 ± 5 nm) with light doses equivalent to 12 and 60 J/cm^2^. The photodynamic effect of Ce6 ethosomes was assessed using the colorimetric MTT assay 24 h later [17].

### 2.6. Determination of Reactive Oxygen Species (ROS) Production

The fluorogenic MitoSOX Red dye specifically targeted to mitochondria in live cells was used to determine superoxide anions by monitoring a fluorescent product with absorption and emission maxima at ~510/580 nm. A431 squamous cell carcinoma cells were treated with 2 µM Ce6 ethosomes for 24 h and thereafter irradiated with the laser at 12 J/cm^2^. At 2 h after PDT application, the collected cells were stained with MitoSOX Red (5 μM) for 30 min and the labelled cells were analyzed by flow cytometry using a BD FACSVerse flow cytometer and BD FACSuite software (BD Biosciences, Heidelberg, Germany) [27].

### 2.7. Analysis of Apoptosis by Active Caspase 3/7

For analysis of active caspase 3/7, 0.2 × 10^6^ A431 squamous cell carcinoma cells per well were seeded in 6-well plates, followed by treatment with Ce6 ethosomes for 24 h. Thereafter, cells were exposed to laser irradiation with a dose of 12 J/cm^2^. At 2 h post PDT, cells were harvested by trypsinization, rinsed with PBS, and incubated with the fluorogenic caspase 3/7 substrate Z-DEVD-R110 (100 µM, Bachem, Bubendorf, Switzerland) in PBS protected from light for 1 h at 37 °C. The cleavage of the substrate by the active caspase 3/7 was analyzed by flow cytometry (Ex/Em 490/525 nm) [27].

### 2.8. 3D Spheroid Culture

Spheroids were established by using the liquid overlay method. First, 50 µL of 1.2% (in 95% ethanol) poly-HEMA (polyhydroxyethylmethacrylate) were added to each well of 96-well round bottom plates and left to dry at 37 °C for two days. A431 squamous cell carcinoma cells or normal human skin BJ fibroblasts were seeded at a density of 5 × 10^4^ cells/well in 200 µL medium DMEM/F-12, 1% streptomycin, and 10% FBS. After 5 days of spheroid formation, the supernatant was cautiously substituted with fresh culture medium prior to treatment [17,28].

### 2.9. Evaluation of PDT Effect of Ce6 Ethosomes Using Light Microscopy and Acid Phosphatase Assay

Squamous cell carcinoma A431 spheroids or normal human skin fibroblast BJ spheroids were incubated with Ce6 ethosomes under standard culture conditions for 24 h. The penetration of fluorescent Ce6 ethosomes (5 µM) was studied for a time range of 60 min using time lapse confocal microscopy (CLSM, LSM 710, Carl Zeiss, Jena, Germany) at 400 nm excitation wavelength and 650 nm emission. Alternatively, the next day after treatment with Ce6 ethosomes (5 µM), spheroids were irradiated with a red laser at 652 ± 5 nm at a light dose of 60 J/cm^2^. After 24 h, the spheroids were carefully transferred to 35 mm Nunc™ glass bottom dishes and recorded under the confocal microscope. Photomicrographs were digitally analyzed with ImageJ 1.43m software (NIH) using the respective color thresholds.

The viability of the squamous cell carcinoma spheroids or normal human skin fibroblast spheroids was evaluated using the activity of the cytosolic acid phosphatases that hydrolyze p-nitrophenyl phosphate to p-nitrophenol in viable cells [28]. Spheroids were washed carefully by replacing 190 µL of the medium with PBS in the 96-well microplates. A total amount of 100 µL per well of the assay buffer (0.1 M sodium acetate, 0.1% Triton X-100 and 2 mg/mL p-nitrophenylphosphate) was added and the plates were incubated for 90 min at 37 °C. Then, 10 µL of 1 N NaOH was added to each well and absorption was measured at 405 nm on a Zenith 200rt microplate reader (Anthos-Labtec, Cambridge, UK).

### 2.10. Histology and Immunohistochemistry

Spheroids were treated with 5 μM of Ce6 ethosomes for 24 h, and thereafter exposed to laser irradiation at a light dose of 12 J/cm^2^ and left for another 24 h. Then, 30–35 spheroids of each group (control, dark, and light controls and Ce6-ethosome-treated group) were collected from the 96-well plates and fixed in 4% buffered formalin. Control and treated spheroids were embedded in a HistoGel proprietary gel (Tissue Culture unit, Pathology department, National Cancer Institute, Cairo University) [29]. Routine sample processing procedures for paraffin embedding were followed, whereby the paraffin blocks of the obtained spheroids were sliced into 3 μm thick sections using a microtome (Microm HM 315, ThermoFisher Scientific, Waltham, MA, USA) and mounted on microscopic slides. The sections were stained with hematoxylin and eosin (HE) in order to detect histopathological changes. Subsequently, both squamous cell carcinoma and normal skin spheroids were immunohistochemically (IHC) stained using antibodies against the proliferation antigen Ki-67 or active caspase 3 (apoptotic cells marker) and examined by light microscopy (Olympus, Saitama, Japan).

### 2.11. Analysis of Squamous Cell Carcinoma Xenografts in CAM Assay

A431 squamous cell carcinoma cells (1.25 × 10^6^ cells/20 mL medium/matrigel, 1:1, *v*/*v*) were applied onto the chick chorioallantoic membrane 9 days after initiation of incubation. After 3 days of incubation at 37.8 °C, the squamous cell carcinoma tumors were topically treated with 20 µL (19 µg, equivalent to a concentration of 0.16 mM and a dose of 950 µg/kg of egg) of Ce6 ethosomes. The following day, the cells were irradiated with 60 J/cm^2^ using a red laser with a wave length of 652 ± 5 nm. On day 13, tumors were collected, fixed in 4% formalin, and imaged using a light microscope. Tumors were then paraffin-embedded and serial sections (5 µm thickness) were mounted onto glass slides. Sections were either histologically analyzed (HE) or were stained with human antiproliferation antigen antibodies (Ki-67) or a terminal deoxynucleotidyl transferase dUTP nick end labelling (TUNEL) kit for the detection of apoptotic cells. Images of the sections were digitally analyzed using Visupac 22.1 software with an Axiophot microscope (Carl Zeiss, Göttingen, Germany) and a MA-3249 CCD camera (Sony, Tokyo, Japan) [27].

### 2.12. Statistical Analysis

Quantitative results are expressed as the mean ± standard error (SD) of at least three independent experiments. Multigroup analysis was performed with the one-way analysis of variance, followed by the Newman–Keuls post hoc test with SigmaPlot software (Systat Software Inc., San Jose, CA, USA). Significance levels were set at * *p* < 0.05, ** *p* < 0.01, and *** *p* < 0.001.

## 3. Results

### 3.1. Characterization of Ce6 Ethosomes

**Figure 1:**
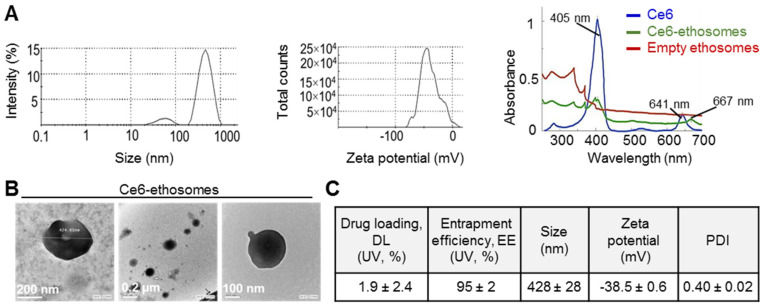
Physicochemical characterization of chlorin e6 (Ce6) ethosomes. (**A**) Mean particle size (left) and zeta potential of Ce6 ethosomes as analyzed by dynamic light scattering and electrophoretic mobility, respectively, in water (0.16 mM). Absorption spectra in water of Ce6 (0.03 mM), Ce6 ethosomes (0.03 mM), and empty ethosomes (15 µg/mL). (**B**) Transmission electron microscope images of Ce6 ethosomes. (**C**) Characterization of Ce6 ethosomes. Drug loading (DL) and entrapment efficiency (EE) were quantified using UV absorption of Ce6; mean particle size, zeta potential, and polydispersity index (PDI) were determined as described in (**A**).

The Ce6 ethosomes are spheric particles measuring about 500 nm with a negative surface charge, which is due to the exposure of negatively charged groups of phospholipids. The absorption spectrum of Ce6 shows a characteristic λ_max_ at about 405 nm and another smaller peak at about 641 nm. Ce6 loaded into ethosomes shows the characteristic λ_max_ for Ce6 at about 405 nm and another smaller slightly shifted peak at about 667 nm. Ce6 in ethosome-loaded form exhibits a decrease in absorption intensity compared to the free form (Figure 1A). Ce6 ethosomes evaluated using TEM showed spherically shaped vesicles with measuring 279–400 nm (Figure 1B). The entrapment efficiency analysis demonstrated the ability of ethosomes to encapsulate the photosensitizer Ce6 with an entrapment efficiency of 95 ± 2%. The drug loading of Ce6 ethosomes was found to be 1.86% ± 2.37%. As a result, an amount of 0.0186 mg of Ce6 was encapsulated per mg of ethosomes. The molar concentrations of Ce6 ethosomes refer to the concentration of Ce6 in ethosomes. The data on the physicochemical characterization of Ce6 ethosomes are summarized in (Figure 1C).

### 3.2. Analysis of Kinetics of Ce6-Induced Singlet Oxygen (^1^O_2_) and ROS Production

**Figure 2:**
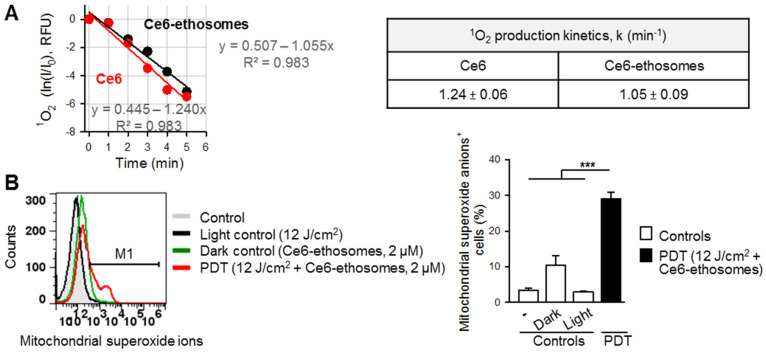
Reactive oxygen species (ROS) generation by Ce6 ethosomes. (**A**) Determination of ^1^O_2_ production kinetics by 0.3 µM of Ce6 (red) and Ce6 ethosomes (black), as analyzed by ADPA sensor fluorescence decay at Ex 378 nm and Em 400–420 nm. The rate constants for ^1^O_2_ production for Ce6 and Ce6 ethosomes are non-significantly different (*p* > 0.05). (**B**) A431 squamous cell carcinoma cells were treated with Ce6 ethosomes (2 µM) for 24 h then irradiated with laser light at 12 J/cm^2^. At 4 h after photodynamic therapy (PDT), the collected cells were stained with 5 μM MitoSOX, a mitochondrial peroxide sensor, and the labelled cells were analyzed by flow cytometry (Ex/Em 490/580 nm). Data are the mean ± SD; *n* = 4, *** *p* < 0.001 vs. controls.

Control samples contained either the singlet oxygen sensor alone and were not irradiated or contained the sensor and Ce6 ethosomes and were not irradiated (dark controls). Additional control samples contained the singlet oxygen sensor and were irradiated by light of doses of 12–60 J/cm^2^ (light controls). The above controls showed minimal photobleaching of the ADPA sensor compared to PDT samples containing either Ce6 or Ce6 ethosomes and exposed to the same light doses (12–60 J/cm^2^). The decrease in ADPA fluorescence that is proportional to singlet oxygen generation is slightly but insignificantly higher in samples containing free Ce6 compared to Ce6 ethosomes (Figure 2A). This shows that loading of Ce6 into biocompatible ethosomes does not significantly decrease the ^1^O_2_ production rate.

Singlet oxygen produced by PDT reaction might cause oxidative damage to biological structures, including mitochondrial membranes. Mitochondria, in turn, are a source of proteins, which can initiate apoptotic cell death [30]. We, therefore, further analyzed the generation of ROS, specifically in the mitochondria of squamous cell carcinoma cells subjected to PDT. Indeed, the cancer cells exhibited a significantly higher production of mitochondrial superoxide in comparison to controls (Figure 2B).

### 3.3. Photodynamic Effect of Ce6 Ethosomes on Squamous Cell Carcinoma Monolayer

**Figure 3:**
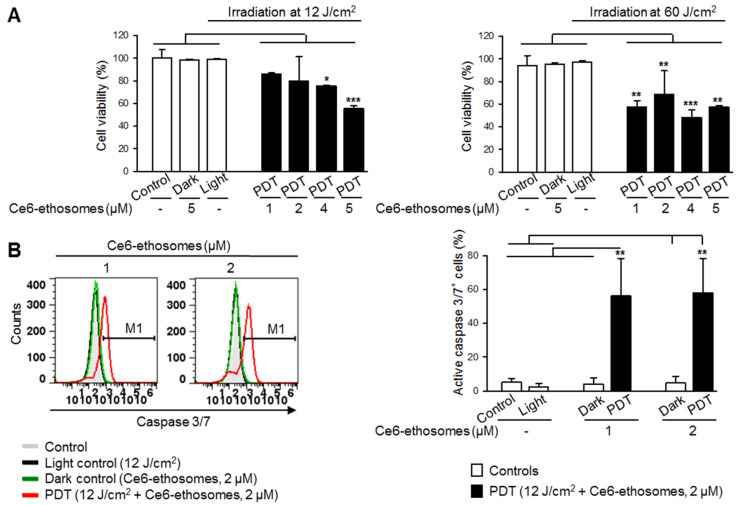
Ce6 ethosomes induce apoptosis in squamous cell carcinoma cells. (**A**) Viability of A431 cells was analyzed by MTT assay after treatment with the indicated concentrations of Ce6 ethosomes for 24 h, followed by light exposure and analysis 24 h later. (**B**) A431 squamous cell carcinoma cells were treated with Ce6 ethosomes (2 µM) as in (**A**) except for 2 h, stained with fluorogenic caspase 3/7 substrate, and analyzed by flow cytometry (Ex/Em 490/525 nm). Data are mean ± SD; *n* = 3, * *p* < 0.05, ** *p* < 0.01, *** *p* < 0.001 vs. controls.

The PDT effect on squamous cell carcinoma cells was evaluated after 24 h incubation with 1–5 µM Ce6 ethosomes after exposure to either 12 or 60 J/cm^2^ light dose. PDT with the lower light dose of 12 J/cm^2^ induced a concentration-dependent decrease of cell viability, with a maximum inhibitory effect of 56% cell viability at 5 µM Ce6 ethosomes. At PDT of 60 J/cm^2^, cell viability was reduced by about 50% without obvious concentration dependency (Figure 3A).

In order to examine if Ce6 ethosomes induce apoptosis in squamous cell carcinoma cells, activation of the executioner caspase 3/7 was analyzed. The caspase 3/7 activity in squamous cell carcinoma cells after PDT was significantly increased in comparison to control samples, suggesting activation of the intrinsic apoptotic pathway (Figure 3B).

### 3.4. Photodynamic Effect of Ce6 ethosomes on Squamous Cell Carcinoma 3D Spheroids

**Figure 4:**
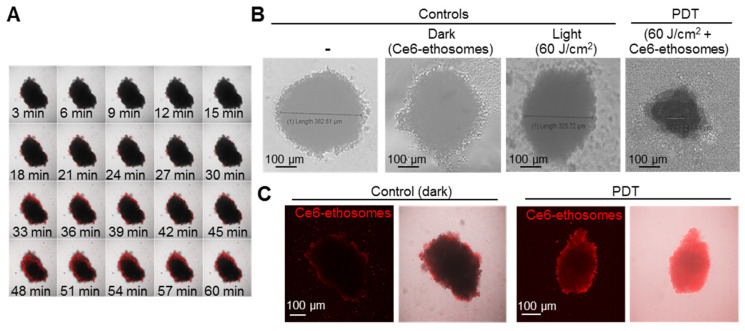
Evaluation of PDT effects of Ce6 ethosomes on human squamous cell carcinoma spheroids. (**A**) Confocal microscopic images of A431 squamous cell carcinoma spheroids treated with 5 µM Ce6 ethosomes (Ex/Em 400/660 nm) for up to 60 min. (**B**) Control (A431 spheroids only), dark control (A431 spheroids treated for 48 h with 5 µM Ce6 ethosomes), and light control (A431 spheroids treated with 60 J/cm^2^ light dose only, analyzed 24 h later) were compared to PDT-treated spheroids (A431 spheroids treated for 24 h with 5 µM Ce6 ethosomes (treated with 60 J/cm^2^ light dose, analyzed 24 h later by light microscopy). (**C**) Fluorescence microscopy of squamous cell carcinoma spheroids treated as in (**B**). The initial magnification was 20×.

The effects of Ce6 ethosomes were further confirmed in squamous cell carcinoma grown in spheroids, which closer resemble tumor growth in vivo. Such squamous cell carcinoma spheroids exhibit a characteristic necrotic core and highly proliferative peripheral tumor outer cells [31]. Light microscopic imaging showed that squamous cell carcinoma spheroids exhibited a round morphology, reaching a plateau diameter of about 350 μm after 5 days of culture. Penetration of Ce6 ethosomes into spheroids was analyzed by confocal microscopy using Ce6 fluorescence. After 60 min, Ce6 ethosomes penetrated to a depth of 5–20 μm, allowing targeting of highly proliferative surface tumor cells by PDT (Figure 4A). PDT of spheroids treated for 24 h with Ce6 ethosomes induced a significant decrease in the spheroid diameter to 240 ± 6 μm compared to control (382 ± 20 μm) (*** *p* < 0.001) and loss of spheroid structural integrity. By contrast, there were no differences in spheroid diameter between different control samples (Figure 4B,C).

**Figure 5:**
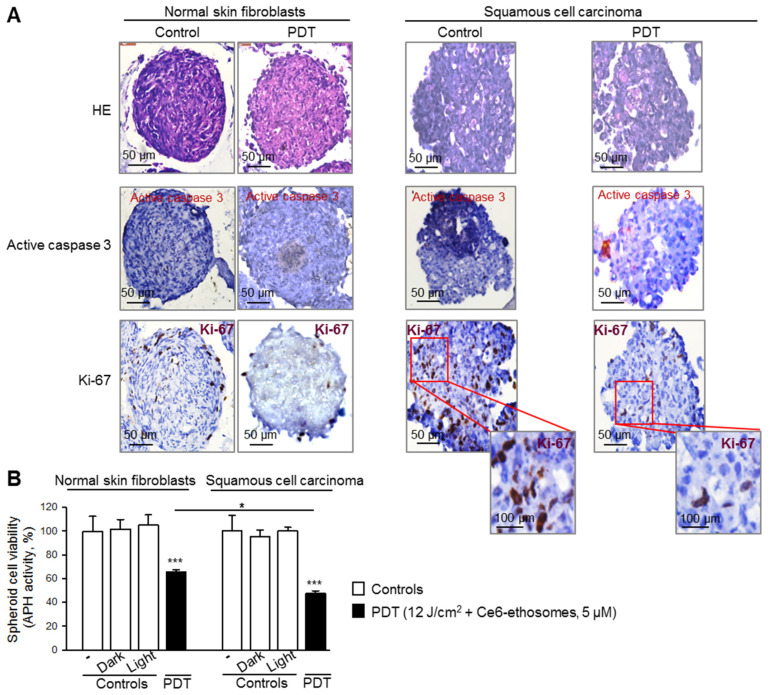
Comparison of PDT effect of Ce6 ethosomes on 3D cultures of normal skin fibroblasts and squamous cell carcinoma. Five-day spheroids were either untreated or PDT-treated (5 µM Ce6 ethosomes for 24 h, then treatment with 12 J/cm^2^ light dose, followed by analysis after 24 h). Controls: spheroids only, including dark control (spheroids treated for 48 h with 5 µM Ce6 ethosomes) and light control (spheroids treated with 12 J/cm^2^ light dose only, analyzed 24 h later). (**A**) Spheroids were fixed, embedded, and cut into 3 µm slices. Staining with antibodies against active caspase 3 indicated induction of apoptosis (red cellular stain); staining with anti-Ki-67 showed proliferating cells (brown nuclear stain); HE, hematoxylin, and eosin. The original magnification was 40x. (**B**) Cell viability was evaluated by analysis of activity of cytosolic acid phosphatases. Data are mean ± SD; *n* = 3, * *p* < 0.05 fibroblasts vs. squamous cell carcinoma, *** *p* < 0.001 vs. respective controls.

In order to analyze how specific the PDT effect of Ce6 ethosomes for cancer cells is, normal skin fibroblasts grown in 3D culture were treated using the same protocol as for A431 squamous cell carcinoma spheroids. Control fibroblast spheroids exhibited a round morphology and a mean diameter of 420 ± 30 μm after 5 days of culture. The photodynamically treated spheroids showed loss of spheroid structural integrity and smaller diameter compared to control spheroids (420 ± 5 μm in control samples vs. 370 ± 10 μm after PDT, * *p* < 0.05) (Figure 5). Immunohistochemical analysis demonstrated that proliferation of fibroblasts and squamous carcinoma cells forming spheroids were likewise reduced, and the cancer cells exhibited some cells positive for active caspase 3, indicating induction of apoptosis (Figure 5A).

The efficacy of PDT application on 3D cultures of normal skin fibroblasts and squamous cell carcinoma was quantified by analysis of the activity of cytosolic acid phosphatases. Despite the toxic effect of PDT on both cultures, there was a significantly higher (about 1.4 times) cytotoxic effect on squamous cell carcinoma spheroids compared to normal skin fibroblast spheroids (Figure 5B).

### 3.5. Photodynamic Effect of Ce6 Ethosomes on Squamous Cell Carcinoma Xenografts

**Figure 6:**
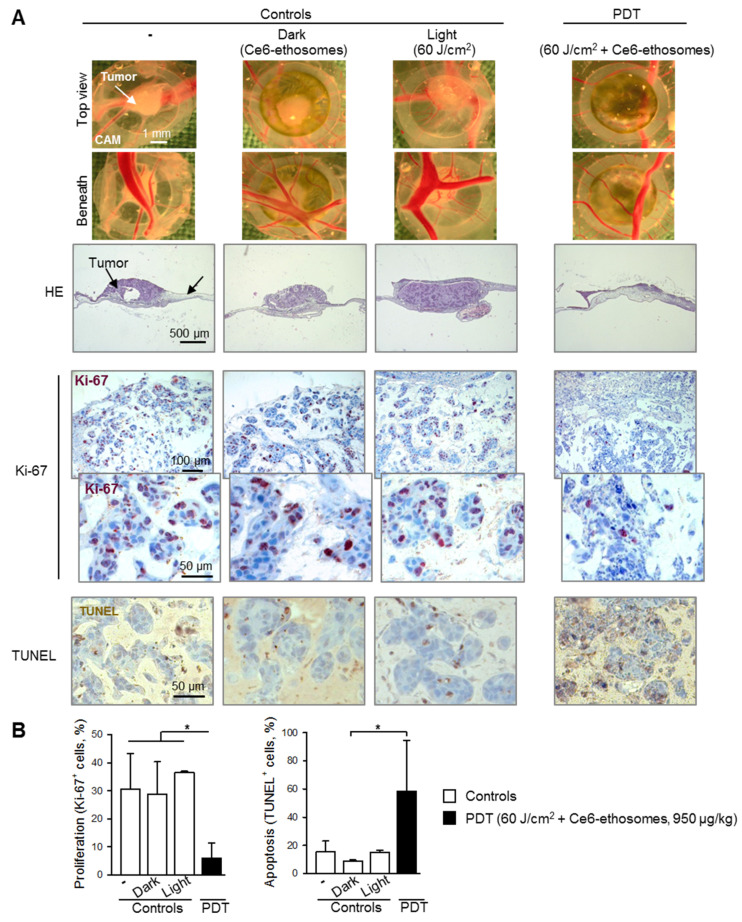
Evaluation of PDT effect of Ce6 ethosomes on human squamous cell carcinoma xenografts. A431 squamous cell carcinoma cells were grafted onto the chorioallantoic membrane (CAM) of fertilized chick eggs. On day 3 after transplantation, tumors were topically treated with 950 µg/kg of Ce6 ethosomes for 24 h. The following day, cells were irradiated with a 60 J/cm^2^ dose of red laser light at 652 ± 5 nm and analyzed 72 h later (PDT). Controls xenografts were treated with solvent only (96 h), including dark control (xenografts treated for 96 h with 950 µg/kg Ce6 ethosomes) and light control (spheroids treated with solvent for 24 h and 60 J/cm^2^ light dose, analyzed 72 h later). After treatment, tumors were collected, fixed, paraffin-embedded, cut into 5 µm slices, and analyzed. (**A**) Representative light microscopic images of squamous cell carcinomas taken after collection (above and below views). HE: hematoxylin and eosin; Ki-67: immunohistochemical analysis of cell proliferation (Ki-67 proliferation marker, brown nuclear stain); apoptosis: TUNEL, brown cellular stain. The original magnification was 200×. (**B**) Quantification of PDT’ effect on cell proliferation and apoptosis of Ce6 ethosomes in human squamous cell carcinoma xenografts. Data are mean ± SD; *n* = 3, * *p* < 0.05 vs. controls.

The PDT effect of Ce6 ethosomes was analyzed in squamous cell carcinoma xenografts in the CAM model. Histopathological analysis showed a reduction in tumor size post PDT and little or no effect on tumors either exposed to laser only (light control) or incubated with Ce6 ethosomes only (dark control) in comparison to untreated control tumors. Light microscopic images taken from the chorioallantoic membrane beneath the tumors showed reduced angiogenesis after PDT (Figure 6A). Immunohistochemical analysis showed that PDT-treated tumors exhibited reduced cell proliferation. In addition, PDT-treated tumors had a significantly increased proportion of apoptotic TUNEL-positive cells (Figure 6A,B).

## 4. Discussion

PDT is a minimally invasive treatment option, which is particularly suitable for superficial cancers such as skin tumors. [8]. Local application of a photosensitizer and light causes cytotoxicity localized at the tumor site, avoiding systemic side effects and minimizing skin scarring if compared to surgical approaches [4]. However, the main concerns associated with many PDT photosensitizers are their poor bioavailability, low selectivity toward malignant cells, and low levels of ROS production in aqueous media [8]. In this respect, the FDA approved photosensitizer, Ce6, which has been used in this study, exhibits several beneficial features—it strongly absorbs light in the red region of the visible spectrum, generates singlet oxygen with a high quantum yield, and can be activated by both light and ultrasound [13]. Moreover, Ce6 accumulates more effectively in tumors compared to healthy tissue, penetrates through cell membranes, and can be cleared rapidly from an organism [13].

In order to increase Ce6 delivery into deeper skin layers, we loaded Ce6 into phospholipid ethosomes. Ethosomes, due to their deformability, have been previously shown to significantly increase skin penetration and cellular uptake of drugs into normal and cancer cells [16,17,32,33]. Due to the presence of surfactant and alcohol, which enhance the fluidity of the phospholipids, ethosomes exhibit remarkable deformability and flexibility [16,34], which could explain their ability to penetrate through several cell layers. Thus, confocal microscopy revealed that Ce6 ethosomes reached the core of a large tumor spheroid measuring 350 nm after 24 h of treatment. Similarly, in our previous studies we have shown enhanced cellular uptake of ethosomes [35] and deep (up to lower dermis) penetration of ethosomes into mouse skin [17]. This suggests that Ce6 ethosomes can be used for invasive squamous cell carcinomas as opposed to treatment, which can only reach superficial tumors in skin.

An additional benefit of biocompatible ethosomes as drug carriers is the significantly reduced systemic toxicity of cytotoxic drugs [34]. Ethosomes enable preparation of water-soluble suspensions of otherwise hydrophobic drugs and increase the stability of such suspensions. Thus, the negative value of the surface charge of Ce6 ethosomes owing to the presence of negatively charged phosphate groups of phospholipids results in better stability in suspensions, as more repulsion occurs between individual particles and their aggregation upon storage is decreased [16].

Incorporation of Ce6 into ethosomes did not significantly alter the ability of Ce6 to generate ^1^O_2_, one of the main destructive species during PDT [4]. This could be attributed to the high fluidity and ultradeformability of the carrier, which made it easy for the ^1^O_2_ produced inside the ethosomes to diffuse out and react with cellular molecules, causing induction of destructive ROS. Indeed, squamous cell carcinoma treated with Ce6 ethosomes followed by light exposure exhibited increased mitochondrial ROS production. Exuberant ROS production might exceed the capacities of the cellular antioxidant system and affect the mitochondrial membrane integrity. The intermembrane space of mitochondria harbors a number of molecules, most notably cytochrome c, which can induce intrinsic apoptosis manifested by caspase 3 activation and DNA fragmentation [36]. PDT treatment of the squamous cell carcinoma monolayer and spheroids preloaded with Ce6 ethosomes induced activation of caspase 3 and tumor cell toxicity, indicating that PDT using Ce6 ethosomes induces intrinsic apoptosis in squamous cell carcinoma.

Importantly, Ce6 ethosomes show selectivity towards skin carcinoma cells compared to normal skin cells. Thus, after PDT, squamous cell carcinoma spheroids exhibited lower proliferation and reduced viability compared to normal skin fibroblasts. This selectivity could be due to differences in ROS susceptibility between normal cells and cancer cells influence cell resistance to PDT. Thus, cancer cells exhibit higher metabolic rates, leading to elevated oxidative stress [37]. The additional increase of ROS levels due to PDT might exceed the limited antioxidant capacities of cancer cells and lead to oxidation and damage of cellular structures [38].

PDT using Ce6 ethosomes was further evaluated in an in vivo xenograft model. This approach allowed investigation of tumor growth in a physiologically relevant environment and analysis of the PDT effect on tumor-induced angiogenesis. PDT-mediated vascular endothelial cell damage has been previously recognized as an important antitumor mechanism of PDT in vivo [39]. PDT using Ce6 ethosomes induced a visible decrease in the number and diameter of blood vessels beneath squamous cell carcinomas, reducing the oxygen and nutrient supply to tumor tissue. In addition, PDT reduced cancer cell proliferation and induced apoptosis. Induction of apoptosis, differing from necrosis, is a beneficial feature for a chemotherapeutic drug, as it does not lead to inflammation or other detrimental consequences in healthy neighbouring tissue [39]. Thus, the increased apoptotic population of tumor cells proved that Ce6-mediated PDT is efficient in the reduction of the vascular network around squamous cell carcinomas and in reducing tumor viability.

In summary, our data show that Ce6 encapsulated into ethosomes might be used for treatment of human skin cancers, such as squamous cell carcinoma.

## 5. Conclusions

In conclusion, photodynamic therapy using chlorin-e6-loaded ethosomes induced ROS production and activation of intrinsic apoptosis, leading to reduced growth in squamous cell carcinoma monolayers, 3D culture, and in vivo xenografts.

The data indicate that squamous cell carcinoma spheroids are more susceptible to photodynamic therapy with chlorin-e6-loaded ethosomes compared to normal skin fibroblast spheroids.

The chlorin-e6-loaded ethosomes might present a convenient formulation of chlorin e6 for clinical applications for the treatment of skin cancers.
